# *SHELXT* – Integrated space-group and crystal-structure determination

**DOI:** 10.1107/S2053273314026370

**Published:** 2015-01-01

**Authors:** George M. Sheldrick

**Affiliations:** a Department of Structural Chemistry, Georg-August Universität Göttingen, Tammannstrasse 4, Göttingen, 37077, Germany

**Keywords:** Patterson superposition, direct methods, dual-space recycling, space-group determination, element assignment

## Abstract

The new computer program *SHELXT* employs a novel dual-space algorithm to solve the phase problem for single-crystal reflection data expanded to the space group *P*1. Missing data are taken into account and the resolution extended if necessary. All space groups in the specified Laue group are tested to find which are consistent with the *P*1 phases. After applying the resulting origin shifts and space-group symmetry, the solutions are subject to further dual-space recycling followed by a peak search and summation of the electron density around each peak. Elements are assigned to give the best fit to the integrated peak densities and if necessary additional elements are considered. An isotropic refinement is followed for non-centrosymmetric space groups by the calculation of a Flack parameter and, if appropriate, inversion of the structure. The structure is assembled to maximize its connectivity and centred optimally in the unit cell. *SHELXT* has already solved many thousand structures with a high success rate, and is optimized for multiprocessor computers. It is, however, unsuitable for severely disordered and twinned structures because it is based on the assumption that the structure consists of atoms.

## Abstract 2

*SHELXT* automates routine small-molecule structure determination starting from single-crystal reflection data, the Laue group and a reasonable guess as to which elements might be present.

## 1. Introduction   

Although crystal structure determination by means of X-ray diffraction has had a major scientific impact for the last 100 years, it still requires the solution of the *crystallographic phase problem*. This problem arises because although methods for measuring the intensities of the diffracted X-rays have made considerable progress during that time, the direct experimental measurement of their relative phases is still only rarely practicable. Small-molecule crystal structures are usually solved by the use of probability relationships involving the phases of the stronger reflections, the so-called direct methods (Sheldrick *et al.*, 2001 ▶; Giacovazzo, 2014 ▶) or more recently by the iterative use of Fourier transforms, *e.g.* dual-space methods such as charge flipping (Oszlányi & Sütő, 2004 ▶; Palatinus, 2013 ▶), in which the phases are constrained by the observed reflection intensities in reciprocal space and by the properties of the electron density in real space.

Before the phase problem can be solved, the usual procedure is to determine the space group of the crystal with the help of the Laue symmetry of the diffraction pattern, the presence or absence of certain reflections (the systematic absences) and statistical tests (*e.g.* to distinguish between centrosymmetric and non-centrosymmetric structures). This space-group determination may be upset by the presence of dominant heavy atoms or by pseudo-symmetry affecting the intensities of certain classes of reflections, and in some cases the space group is ambiguous. For example, the space groups *I*222 and *I*2_1_2_1_2_1_ have the same systematic absences, as do *Pmmn* and two different orientations of *Pmn*2_1_.

**Figure 1:**
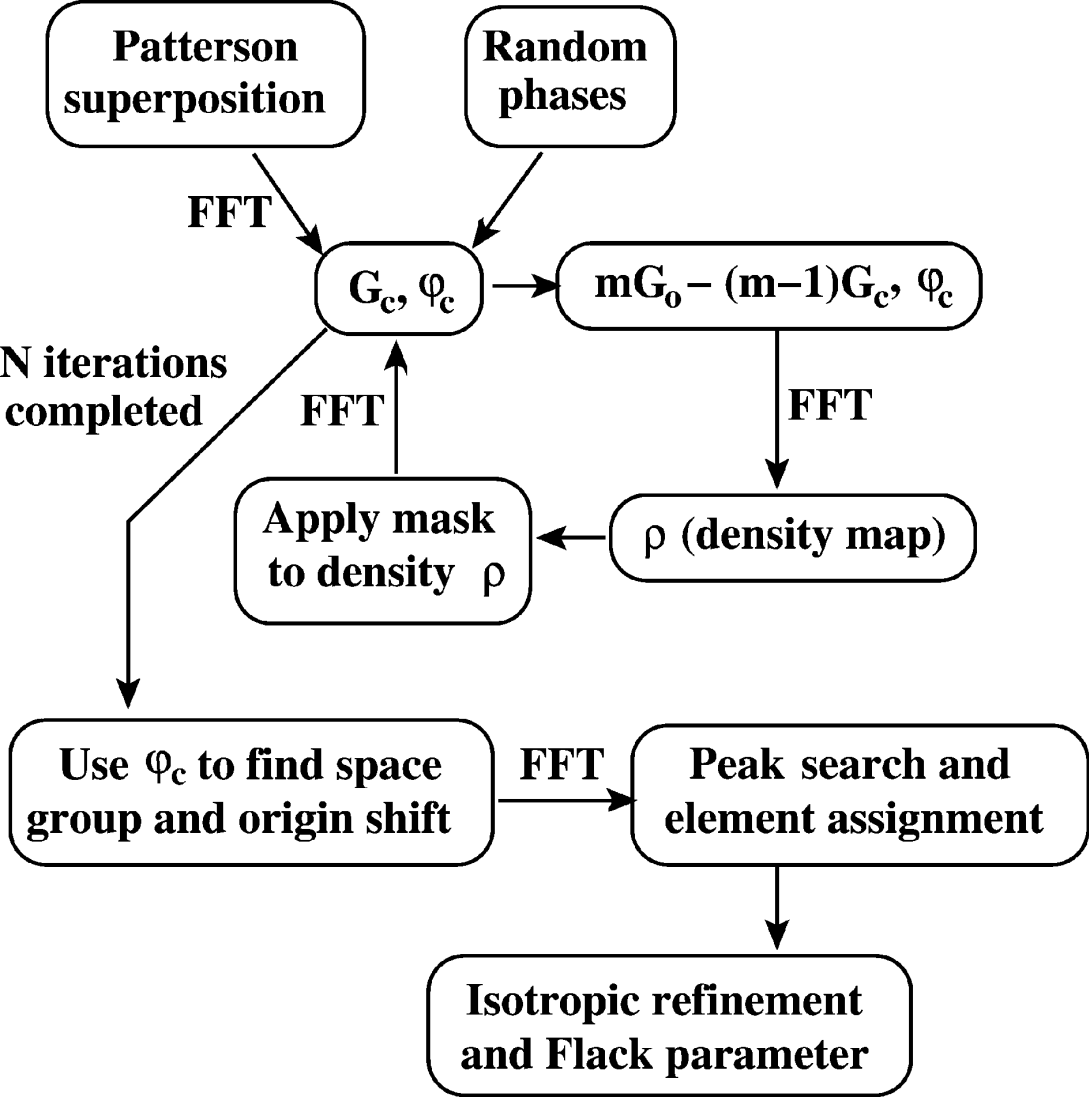
Summary of the *SHELXT* procedure. The dual-space structure solution in *P*1, the space-group assignment and the isotropic refinement are performed in parallel. FFT = Fast Fourier transform. The modified observed and calculated structure factors *G*_o_ and *G*_c_ are discussed in the text and 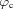 is the phase of *G*_c_.

Many dual-space methods perform at least as well when the data are first expanded to the nominal space group *P*1 (Sheldrick & Gould, 1995 ▶). In this paper ‘*P*1’ will be used to cover the centred triclinic non-centrosymmetric space-group settings such as *C*1 as well; the data do not need to be re-indexed for the primitive cell. After solving the phase problem in *P*1, the space group can be determined using the *P*1 phases (Burla *et al.*, 2000 ▶; Palatinus & van der Lee, 2008 ▶) and this turns out to be a very robust general approach. *SHELXT* also employs this strategy. The systematic absences are not then used for the space-group determination, but all the weak reflections are still useful for identifying the best solution. Fig. 1 ▶ summarizes the course of structure determination using *SHELXT*. The individual stages will now be discussed in detail. The current version of *SHELXT* is intended for single-crystal X-ray data and is not suitable for neutron diffraction data.

## 2. Solving the phase problem for data expanded to space group *P*1   

*SHELXT* reads standard *SHELX* format 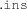 and 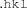 files. It extracts the unit cell, Laue group (but not space group) and the elements that are expected to be present (but not how many atoms of each). A number of options, *e.g.* that all trigonal and hexagonal Laue groups should be considered (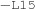), may be specified by command-line switches. A summary of the possible options is output when no filename is given on the *SHELXT* command line and further details are available on the *SHELX* home page.

The data are first merged according to the specified Laue group and then expanded to *P*1. In theory, *SHELXT* could also have been programmed to determine the Laue group, *e.g.* by calculating the *R* values or correlation coefficients when the equivalent reflections are merged. However, the Laue group has to be known to scale the data, which is an essential step for the highly focused beams now common for synchrotrons and laboratory microsources, because the effective volume of the crystal irradiated is different for different reflections and needs to be corrected for. So in practice it is best to determine the Laue group first anyway. Even though programs such as *XPREP* (Bruker AXS, Madison, WI 53711, USA) are no longer required to determine the space group, it is still necessary to identify the correct unit cell and metric symmetry.

### 2.1. Dual-space iteration starting from a Patterson superposition   

The *P*1 dual-space recycling in *SHELXT* may start with random phases, but the default option of starting from a Patterson superposition minimum function (Buerger, 1959 ▶; Sheldrick, 1997 ▶) is usually more effective. Two copies of the sharpened Patterson function, displaced from each other by a strong Patterson vector, are superimposed and the minimum value of the two is calculated at each grid point. The resulting map is used as the initial electron density for the dual-space recycling. In an ideal case it is a double image of the structure consisting of 2*N* peaks, where *N* is the number of unique atoms, but the space-group symmetry has been lost. Since the dual-space recycling is being performed in *P*1 anyway, this is a good start and 2*N* is a significant reduction from the *N* ^2^ peaks in the original Patterson. The subsequent dual-space recycling is performed using the modified structure factors 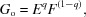
where *E* is the normalized structure factor, and a new density map is calculated by a hybrid difference Fourier synthesis with phases 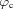 and coefficients
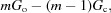
where 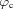 and *G*_c_ are obtained by Fourier transformation of the current map. The default values for *m* and *q* are 3 and 0.5, respectively, but may be changed by the user. Based on experience with other structure-solution programs, *q* should probably be larger for large equal-atom structures and smaller for structures involving heavy atoms (to reduce Fourier ripples), but in practice it is rarely necessary to change the default values.

*SHELXT* adds unmeasured data above and below the resolution limit of the data in the 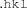 file similar to the *free lunch* method described by Caliandro *et al.* (2005 ▶). This enables structures to be solved at an earlier stage in the data collection and is particularly useful for data collected with diamond-anvil high-pressure cells, with which it is not always possible to collect complete data. It reduces the effects of series-termination errors in the Fourier syntheses, but tends to make the electron-density integration used to assign the element types less reliable.

### 2.2. The random omit procedure   

Omit maps are frequently used in macromolecular crystallography to reduce model bias. A small part of the structure is deleted and the rest is refined to reduce memory effects, then a new difference-density map is generated and interpreted. This concept plays an important role in *SHELXT*, but because no model is available at the *P*1 dual-space stage, it is implemented differently. The following density modification is performed unless otherwise specified by the user. A mask *M*(*x*) is constructed consisting of Gaussian-shaped peaks of unit volume at the positions of the maxima in the electron-density map. A small number of these Gaussian peaks are then deleted from the mask at random, usually every third dual-space cycle, and the new density is obtained by multiplying the original density ρ(*x*) with the mask: 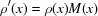
at each grid point *x* in the unit cell. This allows the random omit method to be implemented efficiently using fast Fourier transforms (FFTs) in both directions. Imposing a shape function in this way improves the atomicity of the map. Negative density is truncated to zero, a common theme in phase improvement by density modification (Shiono & Woolfson, 1992 ▶). Compared with charge flipping, the stronger imposition of atomicity probably allows the resolution requirements to be relaxed. On the other hand, charge flipping should be better for the solution of severely disordered or modulated structures, precisely because they are not atomistic!

To decide which *P*1 solution is best, three criteria are considered: (*a*) The correlation coefficient CC between *G*_o_ and *G*_c_, where *G*_c_ are the amplitudes obtained by Fourier back-transformation of the modified electron density. (*b*) The structure factors *G*_c_ are normalized to give *E*_c_ and *R*_weak_ is calculated as the average value of 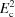 for the 10% of unique reflections (including systematic absences) with the smallest observed normalized structure factors *E* (Burla *et al.*, 2013 ▶). In this way, the weak reflections can still play a decisive role in the structure solution even though they were not used directly to determine the space group. (*c*) The chemical figure of merit CHEM is calculated by performing a peak search and calculating all bond angles involving two distances in the range 1.1 to 1.8 Å. CHEM is the fraction of these angles that lie between 95 and 135° (Langs & Hauptman, 2011 ▶). The combined figure of merit CFOM is given by
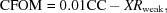
where *X* is 1.0 unless reset by the user. For organic or organo­metallic structures, especially for low resolution or incomplete data, the alternative, 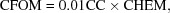
is sometimes better, but this is not the default option because it is not appropriate for inorganic and mineral structures. If CFOM is less than a preset threshold, the program refines further sets of starting phases, increasing the number of iterations each time this is done.

## 3. Using phases to find the origin shift and space group   

The idea of trying all possible space groups in a specified Laue group is also sometimes used in macromolecular crystal structure determination. For example, if the crystal is ortho­rhombic *P*, Laue group *mmm*, and only the Sohncke space groups need to be considered, a molecular-replacement program can be asked to test all eight possibilities. If only one of the eight gives a solution with good figures of merit, both the crystal structure and the space group have been determined! For chemical problems the situation is more interesting, because there are 30 possible orthorhombic *P* space groups and a total of 120 possibilities when different orientations of the axes are taken into account (as in *SHELXT*).

The procedure used in *SHELXT* to find space groups and origin shifts that are consistent with the *P*1 phases is based closely on the methods proposed by Burla *et al.* (2000 ▶) and Palatinus & van der Lee (2008 ▶), so it only needs to be summarized here. For a reflection **h** with *P*1 phase ψ and its *m*th symmetry equivalent **h**_*m*_ = **hR**_*m*_ with *P*1 phase ψ_*m*_, where **R**_*m*_ is a 3 × 3 rotation matrix and **t**_*m*_ is the corresponding translation vector, we define
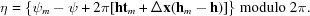
For the correct space group and the correct origin shift Δ**x**, η should be close to zero. To facilitate comparisons, the figure of merit α is defined as the *F* ^2^-weighted sum of η^2^ over all pairs of equivalents for all reflections, normalized so that it should be unity for random phases. α should be as small as possible for the correct combination of space group and origin shift.

*SHELXT* first calculates α for the space group 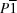; this value is referred to as α_0_. If α_0_ is less than about 0.3, the space group is probably centrosymmetric. For centrosymmetric space groups, the 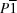 origin shift may be used to place a centre of symmetry on the origin; however, *SHELXT* has to take into account that the space group may possess more than one non-equivalent centre of symmetry. For 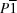, η is calculated with a FFT and for non-centrosymmetric, non-polar space groups a two-dimensional grid search followed by a one-dimensional search is performed to speed up the calculation. The space-group search is performed in parallel for all space groups that need to be tested. Although the solution with the lowest α value is often the correct one, only unlikely solutions with α greater than a specified value (default 0.3) are eliminated before going on to the next stage.

## 4. Assigning chemical elements to the electron-density peaks   

Each solution with a reasonable α value is first subject to ten cycles of density modification in the chosen space group after applying the origin shift. This density modification consists only of averaging the phases of equivalent reflections taking the space-group symmetry into account and resetting negative density to zero. A peak search is then performed, and the density inside a sphere (default radius 0.7 Å) about each peak is summed. It is better to use integrated densities rather than peak heights because the atoms may have different atomic displacement parameters. However, these integrated densities are not on an absolute scale, so the problem is how to set the scale so that they correspond to atomic numbers and the elements can be assigned. *SHELXT* attempts to set the scale as follows, going on to the next test only if the previous tests are negative:

(*a*) If carbon is specified as one of the elements present, the program searches for peaks with similar integrated densities separated from each other by typical C—C distances (*i.e.* between 1.25 and 1.65 Å). If enough are found, the scale is set so that they will have average atomic numbers of 6.

(*b*) If boron is expected, boron cages with distances between 1.65 and 1.8 Å are searched for.

(*c*) A search is made for oxyanions. The oxygen atoms should have similar integrated densities to each other and similar distances to a central atom.

(*d*) If the above tests are negative, it is assumed that the heaviest atom expected corresponds to the peak with the highest integrated density. This can run into trouble if, for example, there is an unexpected bromide or iodide ion in the structure and it has not been possible to fix the scale by one of the above methods.

When the density scale has been found, it is used to assign elements to the remaining atoms. If it then appears that there are high-density peaks that cannot be assigned because only light atoms were expected, chlorine, bromine or iodine atoms are added. Some rudimentary checks are made to ensure that the element assignments are chemically reasonable.

## 5. Isotropic refinement and absolute structure determination   

After the atoms have been assigned, an isotropic refinement is performed using a conjugate-gradient solution of the least-squares normal equations. This is similar to the CGLS refinement in *SHELXL* (Sheldrick, 2008 ▶, 2015 ▶) and is performed in parallel. For non-centrosymmetric space groups this is followed by the determination of the Flack parameter (Flack, 1983 ▶) by the quotient method (Parsons *et al.*, 2013 ▶) and inversion of the structure if the value of the Flack parameter is greater than 0.5. It is thus very likely that the structure determined by *SHELXT* will correspond to the correct absolute structure (so far no examples to the contrary have been reported). If α_0_ is below 0.3 and no atom heavier than scandium is expected, the program stops after finding a plausible centrosymmetric solution. The 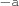 command-line switch may be used to force the program to test all space groups in the assumed Laue group.

## 6. Building the structure   

The following algorithm used to assemble the structure is diabolically simple but almost always builds and clusters the molecules in a way that is instantly recognizable. No covalent radii *etc*. are used, so the algorithm is independent of the element assignments.

(*a*) Generate the SDM (shortest-distance matrix). This is a triangular matrix of the shortest distances between unique atoms, taking symmetry into account.

(*b*) Set a flag to 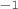 for each unique atom, then change it to 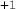 for one atom (it does not matter which).

(*c*) Search the SDM for the shortest distance for which the product of the two flags is 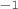. If none, exit.

(*d*) Symmetry transform the atom with flag 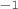 corresponding to this distance so that it is as near as possible to the atom with flag 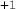, then set its flag to 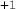.

(*e*) Go to (*c*).

The next stage is to centre the cluster of molecules optimally in the unit cell. This is complicated, but makes extensive use of the tables of alternative origins for the different space groups given in Chapter 3 of Giacovazzo (2014 ▶). For example, for space group 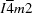 there are four alternative origins (0, 0, 0; 0, 0, ½; ½, 0, ¼; ½, 0, ¾^1^), but for 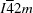 there are only two (0, 0, 0; 0, 0, ½). These are combined with the lattice centring (in this case 0, 0, 0; ½, ½, ½). For polar space groups the optimal position along the polar direction(s) (*e.g.* along the body diagonal of the unit cell for space group *R*3 indexed on a primitive rhombohedral lattice) that minimizes the maximum distance of any atom from the centre of the unit cell is determined.

## 7. Examples   

**Figure 2:**
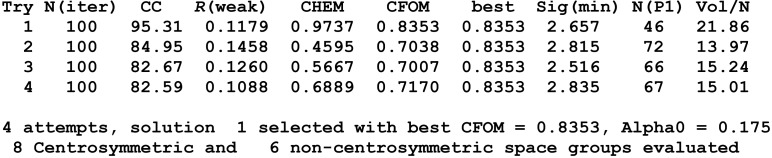
An extract from the 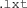 listing file for an organoselenium compound.

The first example is an organoselenium compound (Clegg *et al.*, 1980 ▶) for which an extract from the 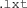 listing file from *SHELXT* is shown in Fig. 2 ▶. Four different Patterson superposition vectors were used by default to start four dual-space structure solution attempts in parallel. This was a good choice because the computer had an Intel i7 processor with four cores. On the evidence of the combined figure of merit CFOM, one of the four (try 1) is a good *P*1 solution. The correlation coefficient CC and the chemical figure of merit CHEM clearly indicate the correct solution, but *R*_weak_ is less clear. *N* is the number of peaks used in the density modification, Sig(min) is the height of peak *N* divided by the r.m.s. (root-mean-square) Fourier map density and Vol/*N* is the volume per peak in Å^3^.

**Figure 3:**
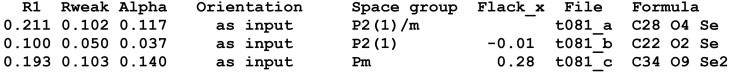
Possible space groups for the organoselenium compound.

The best phase set was then used to search for the space group and three space groups are reported (Fig. 3 ▶); the other 11 space groups tested were rejected because one or more figures of merit were too high. The space group *P*2_1_ is clearly indicated by the values of *R*1, *R*_weak_, α and the Flack parameter, so there can be little doubt that it is correct, and in fact all the atoms are assigned to the correct elements. Note that although α_0_ is less than 0.3, the non-centrosymmetric space groups were searched as well because an atom (Se) heavier than scandium was specified on the 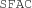 instruction.

**Figure 4:**
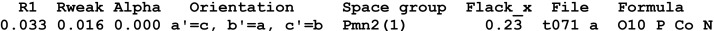
An example where reorientation of the unit cell occurs.

The second example (Müller *et al.*, 2006 ▶) involves a re­orientation of the unit cell. Since two orientations of *Pmn*2_1_ have the same systematic absences, both (and possibly also the centrosymmetric *Pmmn*) would have had to be tried for a conventional structure solution. *SHELXT* finds only one solution and all atoms are correct (Fig. 4 ▶). The Flack parameter is still rather approximate but is sufficient to indicate the correct absolute structure; it improves on anisotropic refinement including the hydrogen atoms.

**Figure 5:**
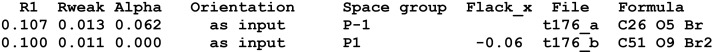
Results for a pseudo-centrosymmetric bromine compound containing a mixture of diastereoisomers.

The third example (Walker *et al.*, 1999 ▶) contains a bromine atom and so the non-centrosymmetric space group *P*1 is also tested, despite the good *R*1 and α values for the centrosymmetric solution (Fig. 5 ▶). In fact, this structure is pseudo-centrosymmetric and contains a mixture of diastereoisomers that imitates a centre of symmetry. The *P*1 solution is completely correct. Both solutions have similar figures of merit because the main difference is the position of one carbon atom that appears to be disordered in 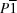 but not *P*1, but the Flack parameter strongly indicates *P*1.

**Figure 6:**
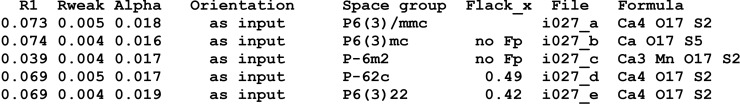
An example showing difficulties that can be encountered when trying to determine the space group.

The last example shows what can go wrong. This structure was published by Barkley *et al.* (2011 ▶) in the non-centrosymmetric space group 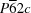, but there are two warning signs: *checkCIF* (Spek, 2009 ▶) detects an inversion centre (a *B* alert) and the Flack parameter is dubious: the current *SHELXL* (Sheldrick, 2015 ▶) gives a value of 0.46 (11). Often a value close to 0.5 indicates a centrosymmetric structure. At first glance, *SHELXT* appears to indicate 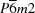 because of a significantly lower *R*1 value. Unfortunately, the Flack parameter cannot be determined by *SHELXT* for this space group because the deposited data had been merged in a different non-centrosymmetric point group (hence ‘
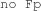
’ in Fig. 6 ▶). However, neither 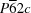 nor 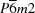 are correct! Basically all the solutions are the same structure and the correct space group is the centrosymmetric *P*6_3_/*mmc* of which all the other space groups are subgroups. The cause of the debacle is that only for 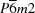 were the elements assigned completely correctly and hence this space group has a lower *R*1 value. For the correct space group *P*6_3_/*mmc* the manganese atom has been incorrectly assigned as calcium. With the correct element assignments all the figures of merit would have been very similar for all the space groups. In such cases the highest-symmetry (centrosymmetric) space group is almost always correct.

## 8. Program development and distribution   

*SHELXT* is compiled with the Intel ifort Fortran compiler using the statically linked MKL library and is particularly suitable for multi-CPU computers. It is available free to academics for the 32- or 64-bit Windows, 32- or 64-bit Linux and 64-bit Mac OS X operating systems. The program may be downloaded as part of the *SHELX* system *via* the *SHELX* home page (http://shelx.uni-ac.gwdg.de/SHELX/), which also provides documentation and other useful information. Users are recommended to view the ‘recent changes’ section on the home page from time to time.

The initial development of *SHELXT* was based on a test databank of about 650 structures, mostly determined in Göttingen, covering a wide range of problems. It has also been tested by more than 200 beta-testers for up to three years, in the course of which several thousand structures were solved (and a few not solved). It is difficult to generalize, but the correct space group was identified in about 97% of cases, and for about half of the structures every atom was located and assigned to the correct element. Most of the remaining structures were basically correct, the most common errors being carbon assigned as nitrogen or *vice versa*. Poor solutions were sometimes obtained when the heavy atoms corresponded to a centrosymmetric substructure but the full structure possessed a lower symmetry. It is always essential to check the element assignments, especially if the program has added extra elements, and also to check for the presence of disordered solvent molecules that may have been missed. The biggest danger is that inexperienced users may assume that the program is always right!

## Footnotes

1 Misprinted as ½, 0, ¼ in Giacovazzo (2014 ▶).
